# Rectal Bioavailability of Amoxicillin from Hollow-Type Suppositories: Effect of Chemical Form of Amoxicillin

**DOI:** 10.3390/pharmaceutics15071865

**Published:** 2023-07-01

**Authors:** Trusha J. Purohit, Satya Amirapu, Zimei Wu, Sara M. Hanning

**Affiliations:** 1 School of Pharmacy, Faculty of Medical and Health Sciences, The University of Auckland, Auckland 1023, New Zealand; t.gor@auckland.ac.nz; 2 Department of Anatomy and Medical Imaging, Faculty of Medical and Health Sciences, The University of Auckland, Auckland 1023, New Zealand; s.amirapu@auckland.ac.nz

**Keywords:** hollow-type suppositories, amoxicillin, rectal drug delivery, bioavailability, tissue tolerance, stability

## Abstract

Rectal drug administration could offer advantages in the delivery of medicines for children by avoiding swallowability issues, improving stability and enabling administration by caregivers. This study aimed to evaluate the rectal bioavailability of hollow-type suppositories (HTS) and understand the effect of two chemical forms of amoxicillin: amoxicillin sodium (AS) or amoxicillin trihydrate (AMT). HTS were prepared by incorporating a lipophilic core containing the antibiotic with a polyethylene glycol (PEG) shell. Formulations were characterised in vitro, and the absolute bioavailability was determined in a rabbit model, while drug–base interactions were evaluated using X-ray diffraction crystallography (XRD), differential scanning calorimetry (DSC) and Fourier transform infrared spectroscopy. The in vitro amoxicillin release from AMT HTS was delayed, taking 27.3 ± 4.9 h to release 50% drug compared with 1.7 h for the AS HTS, likely due to solubility differences between AMT and AS. The presence of orthorhombic AMT and anhydrous AS crystals in respective HTS was confirmed via XRD and DSC. PEG shells were able to protect the drug chemical stability when stored at 25 °C/60% RH. Despite the difference in their in vitro release rates, a similar rectal bioavailability was found in both forms of amoxicillin (absolute bioavailability 68.2 ± 6.6% vs. 72.8 ± 32.2% for AMT HTS and AS HTS, respectively; *p* = 0.9682). Both HTS formulations showed little or no irritation to the rectal mucosa following a single dose.

## 1. Introduction

Rectal administration of amoxicillin could offer advantages over currently available oral and parenteral amoxicillin dosage forms by avoiding swallowability issues, improving stability and enabling administration by caregivers [1]. These formulations could be an attractive treatment option for paediatric patients, especially in low- and middle-income countries (LMICs). Amoxicillin is a broad-spectrum antibiotic recommended for the treatment of infectious diseases. Oral amoxicillin is currently the preferred treatment for fast-breathing pneumonia with no chest indrawing in these regions, but there are limitations to the existing dosage forms [2]. Oral liquid formulations have limited availability in LMICs, require refrigeration and have a short expiry following reconstitution. Children under five years old may have difficulty swallowing oral solid dosage forms such as tablets and capsules. Dispersible tablets are indicated as the preferred dosage form for children in LMICs and overcome some of these challenges [2]. However, they may not be appropriate in all resource-constrained settings, for example, clean water is still required for dispersion.

Alternatives to the oral route of amoxicillin delivery have been explored. For example, hydrogel-forming microneedle arrays have been investigated for transdermal delivery of amoxicillin in paediatric patients. Though minimally invasive, amoxicillin instability was reported due to the moisture and temperature in microneedle arrays [3]. Amoxicillin-containing rectal drug delivery systems have been identified as an alternative paediatric dosage form that is relatively inexpensive to manufacture compared with other non-oral dosage forms, and may be able to overcome these stability challenges by protecting the drug from moisture and high temperatures [4,5,6].

Amoxicillin is available as a trihydrate and sodium salt. Amoxicillin sodium (AS) has an aqueous solubility of 150 mg/mL [7], whereas amoxicillin trihydrate (AMT) has a lower aqueous solubility of 2.7 mg/mL [8]. It has previously been ascertained that AS is absorbed via non-saturated transport from the rectum in a rabbit model [5]. It was also found that the bioavailability of AS was related to the drug release rates from the two types of suppositories, which were formulated with either hydrophilic polyethylene glycol (PEG) 1500:4000 (70:30 *w*/*w*) or lipophilic Suppocire^®^ NA 15 (SNA 15). Although SNA 15 offered greater bioavailability and drug stability than the PEG suppository, its low melting point of 30–36 °C could lead to physical instability if suppositories are exposed to high temperatures [9]. Due to this low melting point, SNA 15 suppositories would need cold chain transport, thus increasing barriers to access and availability, especially in LMICs [10]. This may limit the use of SNA 15 suppositories for the outpatient treatment of pneumonia in these regions. One way to remove the need for cold chain transport without increasing packaging requirements is to develop hollow-type (HT) suppositories, which can accommodate a solid, liquid or semi-solid within a hollow core [1,11,12]. This may improve the physical stability by encapsulating an amoxicillin-loaded SNA 15 core with a polymer shell that can maintain stability in temperatures above the melting point of SNA 15, which is 30–36 °C.

AS is reportedly hygroscopic in nature [13,14], which has the potential to augment rectal irritation by drawing moisture away from the mucosa. AMT is not hygroscopic [13], but the lower aqueous solubility may impact drug transport through the rectal mucosa. Therefore, both AS and AMT suppositories were developed and evaluated in the present study. This study aimed to develop HTS containing 250 mg amoxicillin equivalent using traditional, low-cost suppository excipients in a PEG shell and determine the influence of amoxicillin salt (AS or AMT) on in vitro drug release. The developed suppositories were also evaluated for stability at 25 °C/60% relative humidity (RH) and 37 °C. Finally, the absolute bioavailability of the two types of HTS was evaluated using a previously developed rabbit model [5].

## 2. Materials and Methods

### 2.1. Materials

AS (>87% purity) and AMT (>99% purity) were both purchased from Alfa Aesar by Thermo Fisher Scientific (Haverhill, MA, USA). PEG 1500 and dialysis bags with a molecular weight cut-off (MWCO) of 14,000 Da were purchased from Sigma Aldrich (St. Louis, MO, USA). SNA 15 pellets were a gift from Gattefossé SAS, Saint-Priest, France. Reagent-grade chemicals (acetic acid, sodium acetate, sodium chloride, sodium/potassium dihydrogen phosphate and disodium hydrogen phosphate) were used in the preparation of buffer media and purchased from either Merck or Thermo Fisher Scientific (Auckland, New Zealand). Acetonitrile and methanol used in the chromatographic analysis were high-performance liquid chromatography (HPLC)-grade. Milli-Q water was obtained from a Millipak water dispenser (Millipore, Burlington, MA, USA, 0.22 µm).

### 2.2. Preparation of Amoxicillin HTS

**Figure 1:**
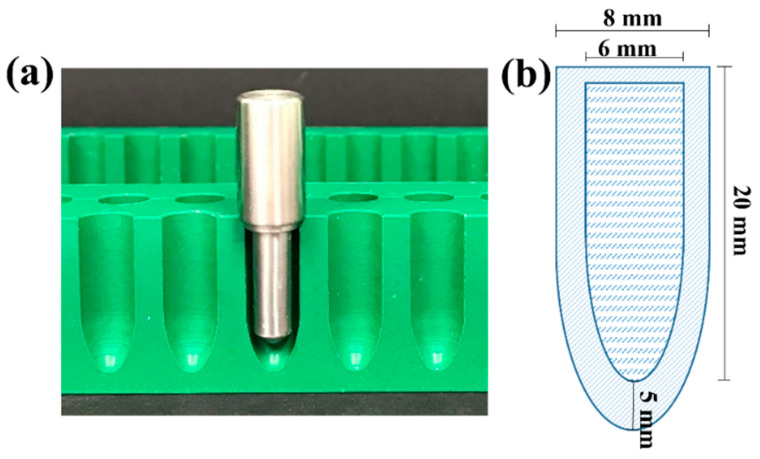
(**a**) The modified hollow-type suppository (HTS) mould showing the stainless-steel pin used for the preparation of PEG shells; and (**b**) a schematic diagram showing the dimensions of the prepared HTS shell and core.

Amoxicillin HTS were prepared as previously described [15], by assembling a PEG 1500 shell and drug-loaded SNA 15 core together using a modified 1 g suppository mould (Figure 1). The shells were prepared via fusion moulding. A PEG base was melted and poured into the mould cavity, then a lubricated stainless-steel pin was immediately placed into the cavity and the PEG was left to solidify. The resulting PEG shells were then carefully separated from the pins and kept in an airtight container until further use.

The warm compression method was used to prepare the core. AS or AMT (equivalent to 250 mg amoxicillin) were weighed individually then combined with 200 mg crushed SNA 15 base per suppository unit. To compensate for losses during the manufacturing process, excess mixture (2%) was prepared. The mixture was compressed into the cavity of a stainless-steel mould pre-warmed to 35 °C. Following solidification for 1 h at room temperature, the compressed cores were taken out of their mould and placed inside the PEG shells. The open end of the shell was then sealed with melted PEG 1500 and allowed to harden. Excess was then removed and the HTS were demoulded.

### 2.3. Characterisation of Amoxicillin HT Suppositories

All suppositories were assessed for the uniformity of shape, size and colour, in accordance with the tests outlined in the British Pharmacopoeia [16].

#### 2.3.1. Uniformity Tests

To determine the weight uniformity, twenty individual units from a batch of both AS HTS and AMT HTS were selected randomly and weighed individually [16]. To determine the content uniformity, AS HTS and AMT HTS (n = 3 for each type) formulations were placed in individual beakers containing 200 mL Milli-Q water at 37 °C. The beaker contents were stirred at 300 RPM for 45 min using a magnetic stirrer, then 1 mL of the sample was withdrawn and filtered using a 0.22 µm syringe filter. Following an appropriate dilution, the samples were quantified using a previously described stability-indicating HPLC method [8].

#### 2.3.2. Hardness of HT Suppositories

The HTS hardness was evaluated using the Erweka hardness tester (Erweka GmbH, Langen, Germany). The tests were conducted to simulate the potential pressure that may be applied to the dosage form during administration or transportation. Three suppositories from each batch were placed in the hardness tester such that the force of the moving plunger was applied on the longitudinal axis of the suppository. The force (kg) required for the HTS to crack or break was considered to represent the hardness.

### 2.4. Characterisation of Drug–Base Interactions

To determine any drug–base interactions causing crystallinity changes in the drug or base, individual samples of each HTS type were characterised using powder X-ray diffraction (XRD), differential scanning calorimetry (DSC) and Fourier transform infrared (FTIR) spectroscopy.

#### 2.4.1. Characterisation of the Core Using Powder X-ray Diffraction (XRD)

Crushed samples of SNA 15 base, AS, AMT, and the drug loaded core of AS HTS and AMT HTS (amoxicillin in SNA 15 base) were compared using XRD (Ultima IV X-ray diffractometer, Rigaku Corporation, Tokyo, Japan). The samples were analysed using Cu Kα radiation (1.54056 Å), with a scattering angle of 5° < 2θ < 45° in a continuous rotation scan mode, with a step size of 0.02θ every 20 s. The diffractometer was operated at a generator tension of 40 KV and current of 30 mA. Diffraction line intensity versus 2θ was plotted and analysed for changes in drug peaks in the presence of SNA 15 base.

#### 2.4.2. Differential Scanning Calorimetry (DSC)

Individual samples of the PEG shell, unmedicated SNA 15 base, AS, AMT, AS HTS and AMT HTS were thermally analysed using a differential scanning calorimeter (TA Q2000, TA instruments, New Castle, DE, USA) equipped with TA-Q Universal Analysis 2000 software. Approximately 5–7 mg sample was precisely weighed and heated from 0–250 °C at a rate of 5 °C/min, with nitrogen gas flowing at 20 mL/min. Interference in the melting behaviour of the HTS shell and the drug loaded core was evaluated for each sample.

#### 2.4.3. Drug-Excipient Interactions Using FTIR

An FTIR spectrometer (Billerica, MA, USA) equipped with a diamond crystal (Bruker Tensor 37, attenuated total reflection—ATR) was used to determine whether there were any drug–excipient interactions. Enough sample to cover the crystal surface was extracted from each batch of the suppositories and analysed using FTIR spectroscopy. The samples were kept directly on the surface of the diamond crystal and were examined from an average of 32 scans with an automatic background subtraction, in the range of 400–4000 cm^−1^ and a resolution of 2 cm^−1^.

### 2.5. In Vitro Drug Release and Kinetics

A USP Type II paddle-type apparatus (Hansen Research SR 8 Plus, Hanson Research, Chatsworth, CA, USA) was used to determine the in vitro release of amoxicillin from HTS. Phosphate-buffered saline (PBS; pH 7.4 at 37 °C) was selected as the dissolution medium. The suppositories were placed in a dialysis bag (MWCO 14,000 Da) in 500 mL PBS (n = 3). The stirring speed of the paddle was kept at 50 RPM and 1 mL sample was withdrawn at 0, 15, 30 and 45 min, then 1, 1.5, 2, 2.5, 3, 4, 5, 7, 9, 24, 27, 30, 48, 52, 60 and 72 h. The samples were diluted as required for the quantitative analysis of amoxicillin, using a previously developed stability-indicating HPLC method [8].

The release profiles of each HTS were compared. The time taken for 50% of amoxicillin to be released from the HTS and similarity factor (*f*_2_) was calculated using DDSolver, a Microsoft Excel add-in program [17]. According to the United States Pharmacopeia, an *f*_2_ value between 50 and 100 indicates similarity in the drug release from different formulations, while a value below 50 indicates a dissimilar drug release from two formulations [18].

The resulting release profiles were fitted to mathematical models to establish the likely in vitro drug release mechanism and understand the impact of drug solubility on release. First-order, Hixson Crowell (Equation (1)), Higuchi (Equation (2)) and Korsmeyer–Peppas (Equation (3)) models were fitted to the drug release profiles using GraphPad prism software 8.2.1 (GraphPad software, Inc., San Diego, CA, USA).

(1) $$Q_{0}^{1/3}-Q_{t}^{1/3}=K_{HC}t$$

where Q_0_ indicates the amount of drug in the suppository at time 0, Q_t_ is the amount of drug released at time t, and K_HC_ (slope) is the Hixon Crowell rate constant.

(2) $$Q_{t}=K_{Hig}\sqrt{t}$$

where K_Hig_ (slope) is the Higuchi rate constant.

(3) $$Q_{t}/Q_{\infty }={K_{KP}t}^{n}$$

where Q_t_/Q_∞_ indicates the fraction of drug released at time *t*, K_KP_ (slope) is the Korsmeyer–Peppas rate constant and n is the release exponent. The value of n indicates different release mechanisms. An n value of 0.45 indicates Fickian diffusion, while 0.45 < n < 0.89 indicates drug release via non-Fickian diffusion [19]. The drug release can be considered case-II transport if n ≥ 0.89.

### 2.6. Stability of Amoxicillin in HTS

HTS were wrapped in aluminium foil, stored in an airtight container, and the physical and chemical stability was evaluated for up to three months. AMT HTS and AS HTS formulations (n = 12 each) were stored at 25 °C/60% RH for three months or until >10% degradation of amoxicillin was observed. A separate set of AMT and AS HTS formulations (n = 4 each) were stored at 37 °C. After three months, the suppositories were assessed for any stability changes using the previously described characterisation techniques [15].

### 2.7. Bioavailability of Amoxicillin in a Rabbit Model

The absolute bioavailability of amoxicillin in AMT HT and AS HT was determined using New Zealand white rabbits of either gender weighing between 3 and 4.2 kg. The rabbits were purchased from a registered supplier and investigations were conducted in accordance with ethical approval from the University of Auckland Animal Ethics Committee (reference number 002012).

#### 2.7.1. Study Design and Sample Analysis

Following a minimum of 5 days for acclimatisation, the rabbits were randomly allocated to receive either 250 mg AMT HTS or AS HTS, or a 2 mL intravenous (IV) AS solution (53 mg/mL) equivalent to 100 mg amoxicillin. After a washout period of 14 days, the rabbits were treated with a different formulation. At the end of the two treatment periods, data were obtained from 3 rabbits per formulation.

Following administration, blood samples were collected at predetermined intervals for up to 8 h, and evaluated using a previously developed stability-indicating HPLC method [8]. Briefly, 950 µL of acetonitrile and 0.1 M acetate buffer (18:1 *v*/*v*) ratio was added to 50 µL of the plasma sample, vortex-mixed thoroughly and centrifuged at 4 °C. The supernatant was then evaporated at 40 °C with a gentle nitrogen stream, reconstituted with 50 µL of mobile phase and analysed using HPLC.

#### 2.7.2. Pharmacokinetics and Data Analysis

The half-life (t_1/2_), area under the drug-concentration–time curve (AUC), area under the moment curve (AUMC), volume of distribution (V_d_) and clearance (Cl) were calculated using Microsoft Excel, based on a non-compartmental pharmacokinetic model [20]. The absolute bioavailability (F) was calculated using Equation (4).

(4) $$F\;\left(\%\right)=\left[\mathrm{AU}C_{0–\infty \left(\mathrm{HTS}\right)}\;\times \;{\mathrm{Dose}}_{\mathrm{IV}}/\mathrm{AU}C_{0\text{–}\infty \;\left(\mathrm{IV}\right)}\;\times \;{\mathrm{Dose}}_{\mathrm{HTS}}\right]\times 100$$

Statistical comparison of the pharmacokinetic data was carried out via a Kruskal–Wallis test (non-parametric one-way analysis of variance (ANOVA)), followed by Dunn’s test for multiple comparisons. All statistical evaluations were carried out using GraphPad Prism 9 software (GraphPad Inc, San Diego, CA, USA). Differences were considered significant if *p* < 0.05.

### 2.8. Rectal Tissue Compatibility

The rabbits were euthanised 24 h after receiving their second treatment, with those that received IV amoxicillin last being included as the controls for tissue histology. Rectal tissue was harvested and fixed in 10% *v*/*v* neutral buffered formalin for 24 h before being transferred to 70% *v*/*v* ethanol and stored at 4 °C, as previously described [5]. The fixed tissues were paraffin-embedded and 5 µm thick sections were cut, then stained with haematoxylin and eosin. The sections were observed under a light microscope for imaging and analysis.

## 3. Results and Discussion

### 3.1. Characterisation of Amoxicillin HTS

**Figure 2:**
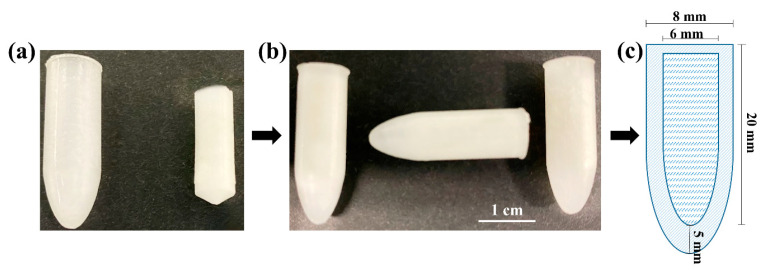
Images representing the components of the HT suppositories: (**a**) separate shell and drug-loaded core; (**b**) HT suppositories with the core loaded inside the shell; (**c**) schematic diagram of HTS, indicating the dimensions of the shell and core.

PEG 1500, with a melting point >45 °C and an acceptable breakpoint, was used for the preparation of drug-loaded HTS, based on the initial characterisation of suppository shells prepared using PEG 1500 and PEG 4000 combinations [15]. HTS were prepared with AS or AMT loaded in lipophilic SNA 15. All suppositories were uniform in size and shape, and slightly off-white in colour (Figure 2).

#### 3.1.1. Uniformity Tests

The average weight of both AS HTS and AMT HTS were 1.17 ± <0.01 g (n = 20 each type). No suppositories deviated by more than 5% of the average weight of the suppositories, indicating a uniformity of weight [16].

The average content of amoxicillin in AS HTS and AMT HTS was 99 ± 2.4% and 109.5 ± 1.9% of the intended 250 mg content, respectively. Both HTS types met the criteria of the British Pharmacopoeia for content uniformity, with an individual suppository content between 75 and 125% of the average content [16].

#### 3.1.2. Hardness Test of HTS

The hardness of AMT HTS was slightly higher (3.7 ± 1.0 kg/cm^2^) than that of AS HTS (3.1 ± 0.3 kg/cm^2^). Hardness was greater than 2 kg/cm^2^ for both suppository types, indicating that the HTS could withstand forces that may be applied during both transport and administration [21].

### 3.2. Characterisation of Drug–Base Interactions

#### 3.2.1. Characterisation of the Drug-Loaded SNA Core Using XRD

**Figure 3:**
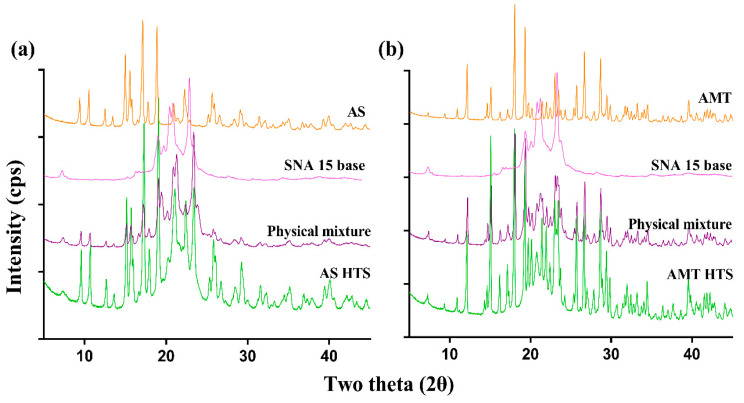
Comparison of XRD patterns of (**a**) AS and (**b**) AMT, their physical mixtures with SNA 15 and the drug-loaded HTS core (both SNA 15).

The XRD patterns suggested that AMT and AS drug powders were crystalline, but diffraction patterns were different (Figure 3). This corresponded to orthorhombic AMT structures and irregular crystal clusters of anhydrous AS [22,23]. Analysis of physical mixture and AS- or AMT-loaded core revealed peaks of crystalline AMT and anhydrous AS, suggesting drug distribution in an amorphous SNA 15 matrix.

#### 3.2.2. Differential Scanning Calorimetry (DSC)

**Figure 4:**
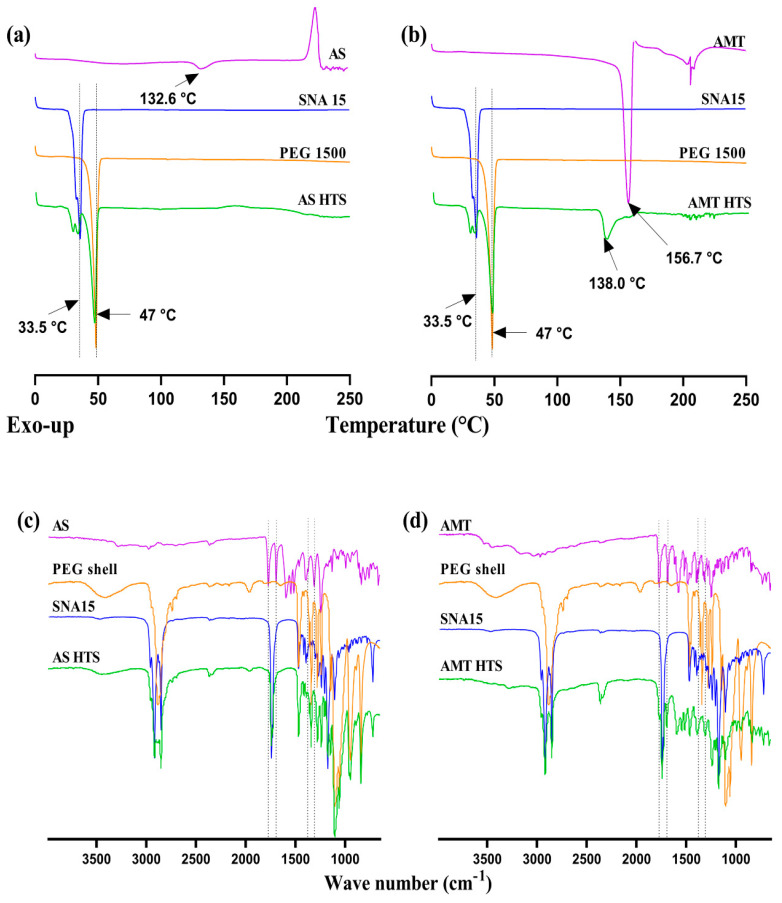
Drug–base interactions shown via DSC analysis, where samples were heated from 0 to 250 °C at a rate of 5 °C/ min of (**a**) AS HTS and (**b**) AMT HTS, along with FTIR spectra of (**c**) AS HTS and (**d**) AMT HTS and individual components.

Thermal analysis using DSC of the individual components and overall HTS are summarised in Figure 4. The thermograms show that both AS and AMT degraded above 230 °C, which is consistent with previous reports [4,13]. A small, broad endothermic peak was observed in the AS sample at 132.6 °C, representing its melting, followed by an exothermic peak at 223.7 °C. In the AMT sample, orthorhombic AMT crystals exhibited a sharp endothermic peak due to melting at 156.7 °C, followed by multiple noise peaks, suggesting drug degradation. The PEG 1500 sample showed an endothermic peak at 47 °C, and SNA 15 displayed several endothermic peaks within the range of 33–36 °C, corresponding to their melting point at 33.6 °C [9].

DSC analysis suggested that the presence of amoxicillin did not influence the melting behaviour of the SNA 15 base (Figure 4). Although AS was distributed as a solid suspension within the SNA 15 matrix, no AS peak was detected in AS HTS. This may be related to the low proportion of AS in the DSC samples, which is supported by the small endothermic peak corresponding to SNA 15 melting and lower enthalpy of the melting of AS. In contrast, AMT HTS showed typical peaks of PEG and SNA 15, as well as a distinct peak at 138 °C likely due to the crystalline rearrangement of AMT. These were followed by multiple peaks within the range of 135–158 °C, indicating possible degradation.

#### 3.2.3. Fourier Transform Infrared Spectroscopy (FTIR)

Qualitative analysis via FTIR corresponded with the DSC analysis, indicating no intermolecular interactions between amoxicillin and the SNA 15 base (Figure 4). Both AS HT and AMT HTS had strong characteristic peaks at 1770 and 1772 cm^−1^ (C=O stretch of the β-lactam ring), 1685, 1693 cm^−1^ (amide I, C=O stretch), 1391, 1377 cm^−1^ (gem-dimethyl CH deformation and phenol OH combination band) and at 1312, 1308 cm^−1^ (fused thiazolidine β-lactam stretch and preliminary amide, C=O stretch), corresponding to the reported values [13].

In the FTIR spectra of AS HT suppositories, the characteristic β-lactam C=O stretching peak at 1770 cm^−1^ was mixed with the characteristic ester C=O stretch peak of SNA 15 base at 1740 cm^−1^. This corresponded with previous findings, where amoxicillin showed combined peaks, but no interaction with the SNA 15 base [5]. In contrast, AMT HTS showed a β-lactam C=O stretching peak at 1772 cm^−1^, as well as amide I C=O stretching peak at 1693 cm^–1^. The peaks in the fingerprint region were found merged with the peaks of the PEG 1500 or SNA 15 bases used in the preparation of the shell and core of both types of HT suppositories.

### 3.3. In Vitro Drug Release

**Figure 5:**
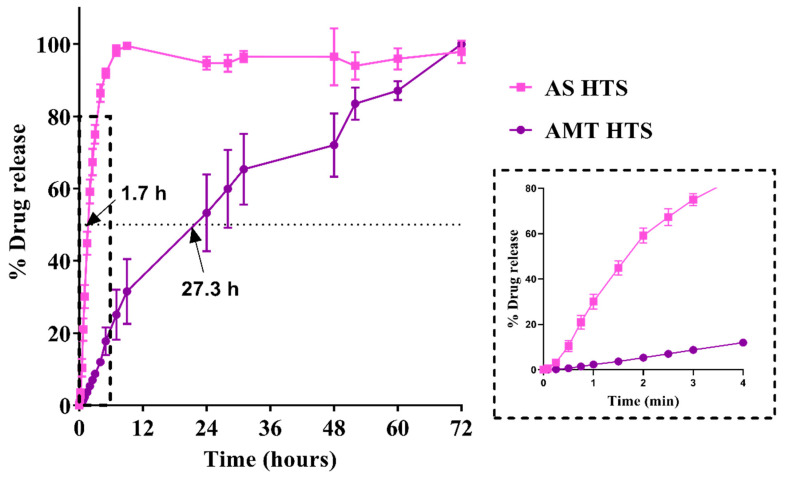
In vitro drug release from AS HTS (pink line) and AMT HTS (purple line), with differences in drug release in the first 4 h highlighted (inset). Data are presented as the mean ± SD of n = 3 suppositories. Dotted line represents the mean dissolution time (MDT).

Different drug release profiles were observed in AS HTS compared with AMT HTS (Figure 5), with a similarity factor *f*_2_ of 18.1. This value is smaller than the minimum threshold of 50 for being similar [18]. A large difference (25.6 h) in the mean dissolution time (MDT) between AS HTS and AMT HTS (1.7 h vs. 27.3 h) was observed due to the difference in their aqueous solubility.

Despite both AS and AMT existing as solid suspensions within a matrix of SNA 15, nearly 80% of amoxicillin was released within 4 h from AS HTS compared with only 12% amoxicillin released from AMT HTS over the same period (Figure 5, inset). Melting, then spreading of the SNA 15 base may have increased the surface area for drug dissolution. However, the low aqueous solubility of AMT may have hindered drug release in the AMT HTS, which can also be seen in the time-lapse images during the dissolution study (Supplementary Figure S1).

The release profile of AMT HTS fitted the Higuchi model, whereas AS HTS fitted the first-order release model, as summarised in Table 1. The Higuchi model describes drug release from a matrix system following Fickian diffusion, whereby the dissolution of the base matrix leads to concentration-dependent drug release [24]. It follows that AMT particles existed in a suppository base matrix where drug release can be attributed to the velocity of dissolution rather than diffusion from the dialysis bag. As AMT is poorly water-soluble (2.7 mg/mL) [8], the arrangement of AMT as indicated by XRD, might have contributed to the slow drug dissolution and release. Furthermore, the presence of three water molecules in the trihydrate form of amoxicillin has a tendency to result in increased hardness [25,26]. This was also reflected in the hardness test and could have possibly resulted in the delayed drug release from AMT HT suppositories.

The first-order model best described AS HT release (R^2^ = 0.9930). This is likely due to the high aqueous solubility of AS (150 mg/mL in water at 25 °C [7]), resulting in the rapid dissolution of particles and release of the drug (Noyes–Whitney theory).

### 3.4. Stability of HTS

After three months, the suppositories stored at 25 °C/60% RH showed no physical signs of melting, whereas softening was observed in the PEG shell of the suppositories stored at 37 °C. However, when exposed to room temperature conditions, the shells regained their hardness within minutes. There was a subtle colour change observed at the top of AS HTS stored at 25 °C/60% RH, whereas the inner core showed no colour change (Supplementary Figure S2). In contrast, the surface of the AMT HTS core changed from white to yellow in colour, although no colour change was observed inside the core upon breaking.

All HTS stored at 37 °C exhibited a colour change after 30 days, with possible hydrolysis of amoxicillin present on the outer surface of the core. This may be the result of the hygroscopic nature of PEG [27,28]. Heat entrapment within the shell when the core and shell were manually assembled may also have led to the degradation of amoxicillin on the surface of the core.

PEG 1500 started to lose its integrity around 36–37 °C, despite having a melting point of about 47 °C. PEG 1500 is a waxy polymer, so it starts to soften before it melts completely between 44 and 49 °C [27]. Exposure at 37 °C for prolonged periods may have led to molecular changes in the PEG chain (for example, increased movement of molecules) and the subsequent softening of the PEG 1500 shell [29]. Although the PEG shells softened at 37 °C, no direct relation of the glass transition temperature to structural firmness was observed [30]. This is a limitation to consider of PEG 1500 shells in protecting the drug-loaded core.

#### 3.4.1. Change in Hardness: Effect of Amoxicillin Form

After three months of storage at 25 °C/60% RH, the hardness increased by 1.3 kg and 1.1 kg in AMT HTS and AS HTS, respectively. In addition, AS HTS hardness increased when stored at 37 °C. The increase in hardness of conventional suppositories may be linked to the rancidity of the lipophilic base upon storage [6]. Hydrates present in the drug form reportedly affect the mechanical strength of pharmaceutical formulations over time [25,26,31,32]. The hardness of HTS was not expected to change over time and the PEG shell was intended to protect against any physical or chemical changes to the amoxicillin-loaded core, so further development is needed to address this.

No significant changes in the thermal properties of the suppositories were observed over time (Supplementary Figure S3a,b), despite the noticeable colour change. In line with DSC findings, no intermolecular interactions were observed from the FTIR spectra. The comparative spectra are included in the Supplementary Data File.

#### 3.4.2. Content of Amoxicillin over Time

Despite the observed colour change in HTS stored at 25 °C and 60% RH, an average of 98.3 ± 1.7% and 102.1 ± 1.8% amoxicillin remained after three months compared with that at time 0 in AS HTS and AMT HTS, respectively. Although a small variation in content after 3 months was observed, this satisfies the requirements for chemical stability according to the United States Pharmacopoeia [33]. Following storage at 37 °C, only AMT HTS met the pharmacopoeia definition of stability, with 100 ± 0.8% amoxicillin remaining after 30 days, whereas AS HT suppositories, with 79.2 ± 8.8% drug remaining, did not. Tests were not continued beyond 30 days due to physical changes in colour and integrity of the PEG shells.

Although amoxicillin content was greater than 98% in both AS HT and AMT HTS stored at 25 °C/60% RH after 3 months, the shells did not prevent colour change likely due to surface hydrolysis of the drug particles. Therefore, further improvements in the preparation of shells and packaging of the suppositories are required to improve the stability of the HT suppositories.

### 3.5. Absolute Bioavailability in Rabbits

**Figure 6:**
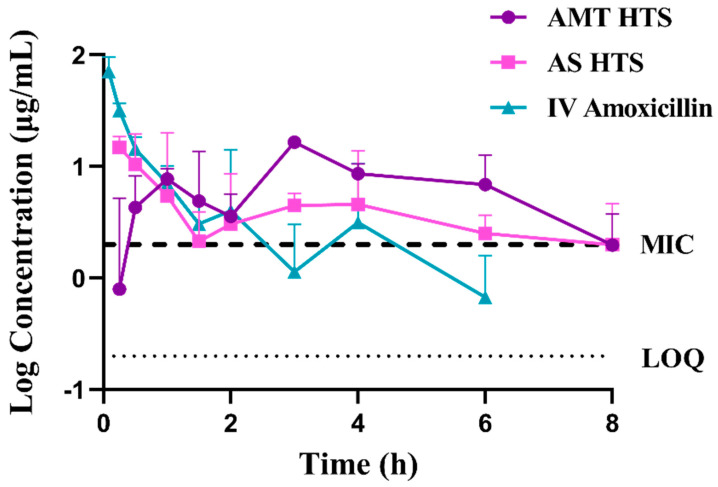
Amoxicillin log plasma concentration versus time profile following rectal administration of AMT HTS and AS HTS (equivalent to 250 mg amoxicillin) compared with amoxicillin IV injection at a dose of 100 mg per rabbit. All data have been presented as the mean ± SD (n = 3 rabbits per treatment). The dashed line shows the minimum inhibitory concentration (MIC) of amoxicillin for *S. pneumoniae* (2 µg/mL), and the dotted line shows the limit of quantification (LoQ) of the HPLC assay.

The drug plasma concentration versus time profiles are shown in Figure 6, and detailed PK parameters are summarised in Table 2. The absolute bioavailability of AMT HTS and AS HTS was found to be 68.2 ± 6.6% and 72.8 ± 32.2%, respectively (*p* = 0.9682). Statistically nonsignificant differences were observed in the AUC values between the two drug forms (*p* = 0.539), as well as each salt form compared with IV amoxicillin (*p* > 0.22). However, large individual variation, particularly within the AS group, and the small sample size could both contribute to the non-significant differences. The high solubility of AS was expected to lead to rapid drug release in vivo despite the small volume of rectal fluid (1–3 mL in humans [1,34]), and a subsequent higher bioavailability. The thin layer of PEG shell was expected to rapidly dissolve and not impact the bioavailability of AS from the SNA 15 core [5].

As previously observed with conventional AS suppositories [5], rapid drug absorption from AS HT was observed with T_max1_ at 0.5 ± 0.4 h, followed by T_max2_ at 3.3 ± 1.2 h. On the contrary, drug absorption from AMT HT was slow, with the first T_max1_ at 1.3 ± 0.3 h. Interestingly, both HTS types showed a double-peak plasma drug concentration profile despite the disparity of the drug forms and their in vitro release patterns. This phenomenon has been reported previously with AS in conventional suppositories prepared with PEG or SNA 15 base [5]. Additionally, the blood plasma concentration of 250 mg amoxicillin was above the minimum inhibitory concentration (MIC) for *Streptococcus pneumoniae* at 2 µg/mL. The mean residence time (MRT) of AMT HTS was longer than AS HTS (96.8 ± 0.4 h and 5.4 ± 0.9 h, respectively). Both were more than five-fold longer than that following IV administration (1.05 ± 0.83 h). Also, the mean absorption time (MAT) of AS HTS was 1.5 h longer than that of AMT HTS (6.19 ± 0.40 vs. 4.72 ± 0.88 h, *p* = 0.0570), attributed to the rapid drug release form AS HT. This confirmed the extended exposure of the antibiotic in vivo, which is specifically beneficial for time-dependent antibiotics like amoxicillin, where the time that the antibiotic is at a plasma concentration above the MIC is important for the antimicrobial effect. Both HTS indicated a steady and consistent release of amoxicillin, suggesting treatment efficacy of amoxicillin following rectal administration. However, the difference in MAT was non-significant, possibly due to the small sample size and large individual variation. Unfortunately, rabbits were not readily available at the time of this study, so a larger sample size (ideally n = 5 as per previous work [5]) was not possible. In addition, one rabbit passed away during the washout period of this study. An investigation by the veterinary team concluded that this was not related to the intervention. Future research should focus on obtaining data from a larger sample size.

The delay in the drug release of AMT observed in vitro compared with AS was not reflected in the in vivo findings. This could be due to a lack of ideal sink conditions during the in vitro release study, which slowed down the drug release from AMT HTS. Rabbits were selected for the present study because they have similar rectal physiology to humans [35,36]. Therefore, it would be expected that similarities in bioavailability would be observed if the dosage forms were to be used in the intended paediatric population. However, further evaluation is needed, including further development of an in vitro model that predicts in vivo findings more accurately before these formulations are ready for human trials.

### 3.6. Rectal Tissue Compatibility

**Figure 7:**
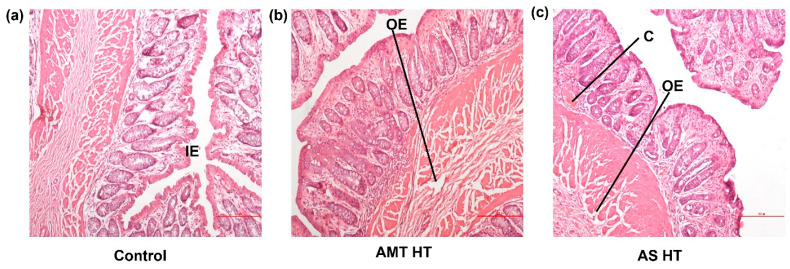
Histological evaluation of rectal tissue irritability of (**a**) the control (IV group with no HTS exposure), compared with (**b**) AMT HTS and (**c**) AS HTS. C—congestion, IE—intact epithelium, OE—oedema (scale bar: 500 px).

Upon a microscopic evaluation of the rectal tissues, minor signs of irritation to the rectal mucosa were observed in the tissue samples collected from all rabbits (Figure 7). Both AMT HTS and AS HTS showed signs of blood vessel congestion and oedema in the lamina propria and mucosal layers. This may be due to the PEG shell, or the exposure of the amoxicillin core to the rectal mucosa upon the dissolution of the shell. A PEG base is known to draw moisture from the surrounding tissues, causing rectal irritation [37], which was also observed previously [5]. A recent report has also suggested mild irritation in the presence of AMT when tested using a slug mucosal assay [4]. Although PEG shells showed minimal irritation to the rectal tissue harvested after 24 h, the anorectal region of paediatric patients may not be fully developed. The clinical effect of this irritation and the effect of repeated exposure should be considered in future work.

## 4. Conclusions

Amoxicillin HTS were developed by loading two chemical forms of amoxicillin, AMT or AS, in a lipophilic SNA 15 core, which was then encapsulated within a PEG 1500 shell. Thermal analysis and FTIR spectroscopy implied no change in the melting behaviour of the suppository bases, and no intermolecular interactions in the formulations. The water-soluble PEG shells promoted a fast exposure of the drug which was loaded in a low-melting-point core to favour drug release. The PEG shells were found to protect the amoxicillin loaded in the lipophilic core when stored at 25 °C/60% RH, with >98% amoxicillin remaining after three months, regardless of the chemical form of amoxicillin. However, this protection was not sufficient when the storage temperature increased to 37 °C. Despite the large difference within the in vitro release profiles of the two types of suppositories, the absolute bioavailability in vivo was similar for the two formulations (68.2 ± 6.6% and 72.8 ± 32.2% for AMT HT and AS HT, respectively). These evaluations indicate the potential for amoxicillin HTS and have highlighted areas for further formulation development.

## Figures and Tables

**Table 1:** Summary of release kinetics of amoxicillin from AS HT and AMT HT suppositories.

Model	AMT HT			AS HT		
	R^2^	Slope	Intercept	R^2^	Slope	Intercept
First-order	0.6869	−0.0255	2.0870	0.9930 **	−0.2533	2.0890
Hixson Crowell	0.9336	0.0458	0.0145	0.9748	0.4629	0.1072
Higuchi	0.9804 **	12.4800	8.5010	0.9405	41.5000	7.7850
Korsmeyer Peppas ^#^	0.9519	1.0200	0.2977	0.6049	1.0610	1.2080

^#^ n = 0.2977 and 1.2080 for AMT HTS and AS HTS, respectively. ** The best model to describe the release kinetics.

**Table 2:** PK parameters of amoxicillin following rectal administration of AMT HTS and AS HTS (equivalent to 250 mg amoxicillin) compared with an IV injection (at a dose of 100 mg per rabbit) in rabbits. Data are presented as the mean ± SD; n = 3 rabbits per treatment. No statistically significant differences were observed between the three formulations.

PK Parameter	IV Amoxicillin (n = 3)	AMT HTS (n = 3)	AS HTS (n = 3)
AUC_0–8h_ (μg/mL·h)	33.95 ± 11.34	56.67 ± 7.45	39.97 ± 17.92
AUC_0–∞_ (μg/mL·h^2^)	36.09 ± 13.35	63.43 ± 6.09	213.96 ± 99.88
T_max1_ (h)	-	1.33 ± 0.29	0.50 ± 0.43
T_max2_ (h)	-	3.00 ± 0.00	3.33 ± 1.15
C_max1_ (μg/mL)	73.40 ± 20.48	9.24 ± 1.60	16.89 ± 6.23
C_max2_ (μg/mL)	-	16.57 ± 1.54	9.22 ± 2.64
t_1/2_ (h)	1.41 ± 0.49	1.86 ± 0.44	3.92 ± 3.02
MRT (h)	1.05 ± 0.83	6.83 ± 0.40	5.36 ± 0.88
MAT (h)	-	6.19 ± 0.40	4.72 ± 0.88
F (%)	100	68.25 ± 6.55	72.75 ± 32.19
V_d_ (L)	5.82 ± 1.46	10.64 ± 2.43	2.22 ± 29.72
Cl (L/h)	3.07 ± 1.24	3.97 ± 0.40	4.82 ± 2.53

“-” means not applicable.

## Data Availability

The data presented in this study are available upon reasonable request from the corresponding authors.

## Supplementary Materials

The following supporting information can be downloaded at: https://www.mdpi.com/article/10.3390/pharmaceutics15071865/s1, Figure S1: Time lapse images captured during the drug release study up to 72 h showing drug release from hollow type suppositories (HTS) kept in a dialysis bag (MWCO 14000 Da) containing PBS, pH 7.4 at 37 °C. (a) AS HTS and (b) AMT HTS. Low aqueous solubility of AMT led to delayed drug release from AMT HTS; Figure S2: Images showing different degrees of colour change in HTS stored at 25 °C, 60% RH after three months. (a) AS HTS and (b) AMT HTS (c) An image showing colour change only at the top end of the HT suppositories. Red arrows indicate the location of colour change in the HT suppositories; Figure S3: Stability of amoxicillin HT suppositories evaluated with DSC (a,b) and FTIR (c,d). DSC analysis of (a) AS- and (b) AMT HT suppositories. The HT suppositories were kept at 25 °C, 60% RH and 37 °C. The samples were heated from 0–250 °C at the rate of 5 °C/min. Intermolecular interaction analysis of (c) AS- and (d) AMT HT suppositories using FTIR. Dotted line indicates β-lactam peak at 1772 cm^−1^; Figure S4: Amoxicillin plasma concentration versus time profile following rectal administration of AMT and AS containing HTS compared with amoxicillin IV injection at a dose of 100 mg per rabbit. All data have been presented as mean ± SD (n = 3). The dashed line shows the minimum inhibitory concentration (MIC) of amoxicillin for S. pneumoniae (2 µg/mL), and the dotted line shows the limit of quantification (LoQ) of the HPLC assay; Figure S5: PK profiles of individual rabbits following the administration of; 100 mg IV solution (a–c), 250 mg AMT HTS (d–f) and 250 mg AS HTS (g–i).
