# Supplementary material for: Rectal Bioavailability of Amoxicillin from Hollow-Type Suppositories: Effect of Chemical Form of Amoxicillin
Source: Pharmaceutics. 2023 Jul 1;15(7):1865. doi: 10.3390/pharmaceutics15071865 (PMC10383505; doi:10.3390/pharmaceutics15071865)
Supplement: Supplementary file 1 [file pharmaceutics-15-01865-s001.zip › pharmaceutics-2397119-supplementary.pdf]

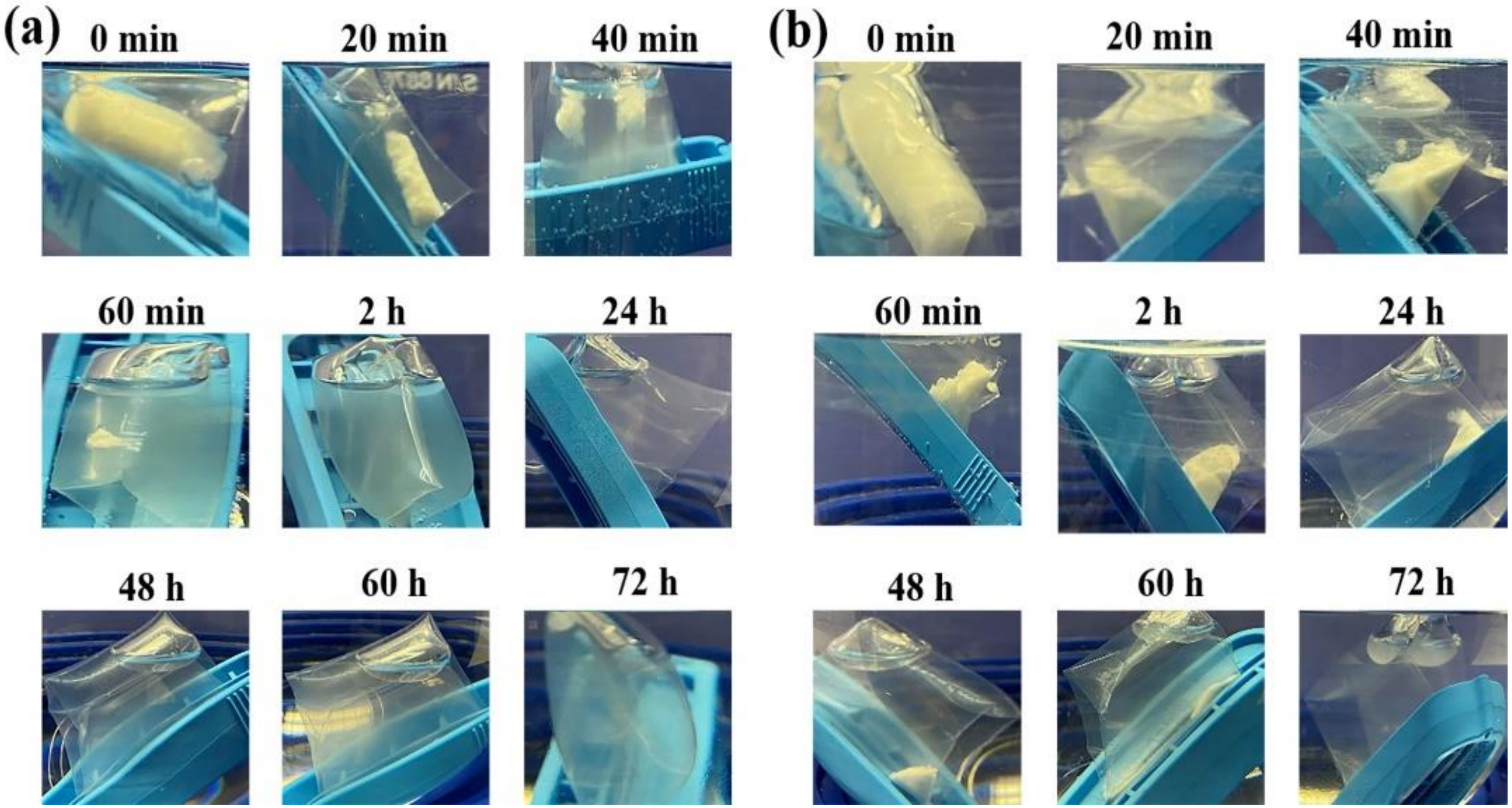

Figure S1. Time lapse images captured during the drug release study up to 72 h showing drug release from hollow type suppositories (HTS) kept in a dialysis bag (MWCO 14000 Da) containing PBS, pH 7.4 at 37 °C, where (a) amoxicillin sodium (AS) HTS and (b) amoxicillin trihydrate (AMT) HTS. Low aqueous solubility of AMT led to delayed drug release from AMT HTS.

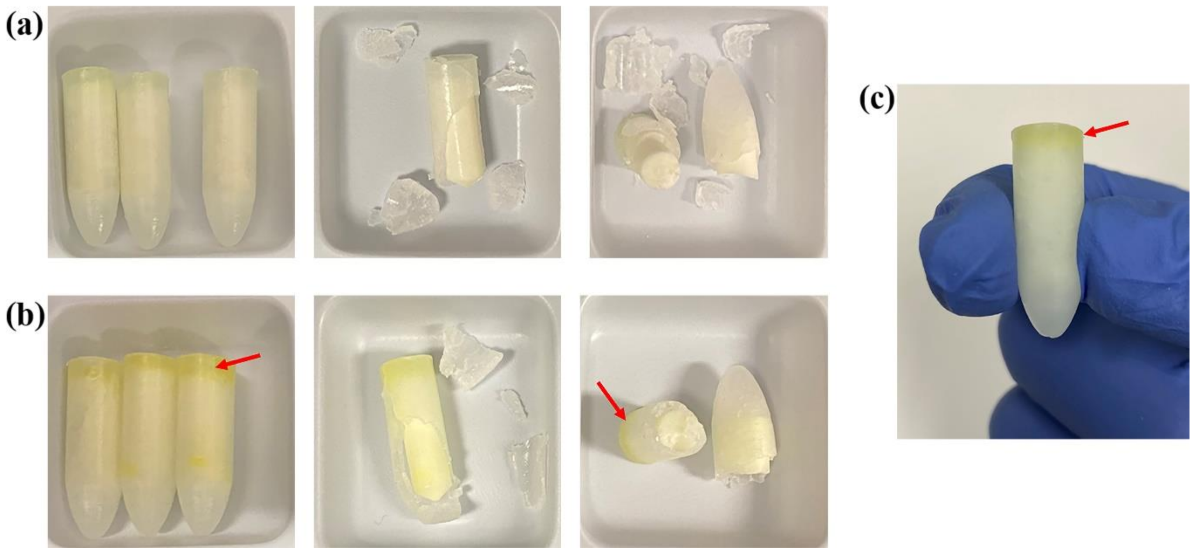

Figure S2. Images showing different degrees of colour change in hollow type suppositories (HTS) stored at 25 °C, 60% RH after three months, where (a) amoxicillin sodium HTS, (b) amoxicillin trihydrate HTS and (c) an image showing the colour change at the top end of the

HTS. Red arrows indicate the location of colour change in the HTS.

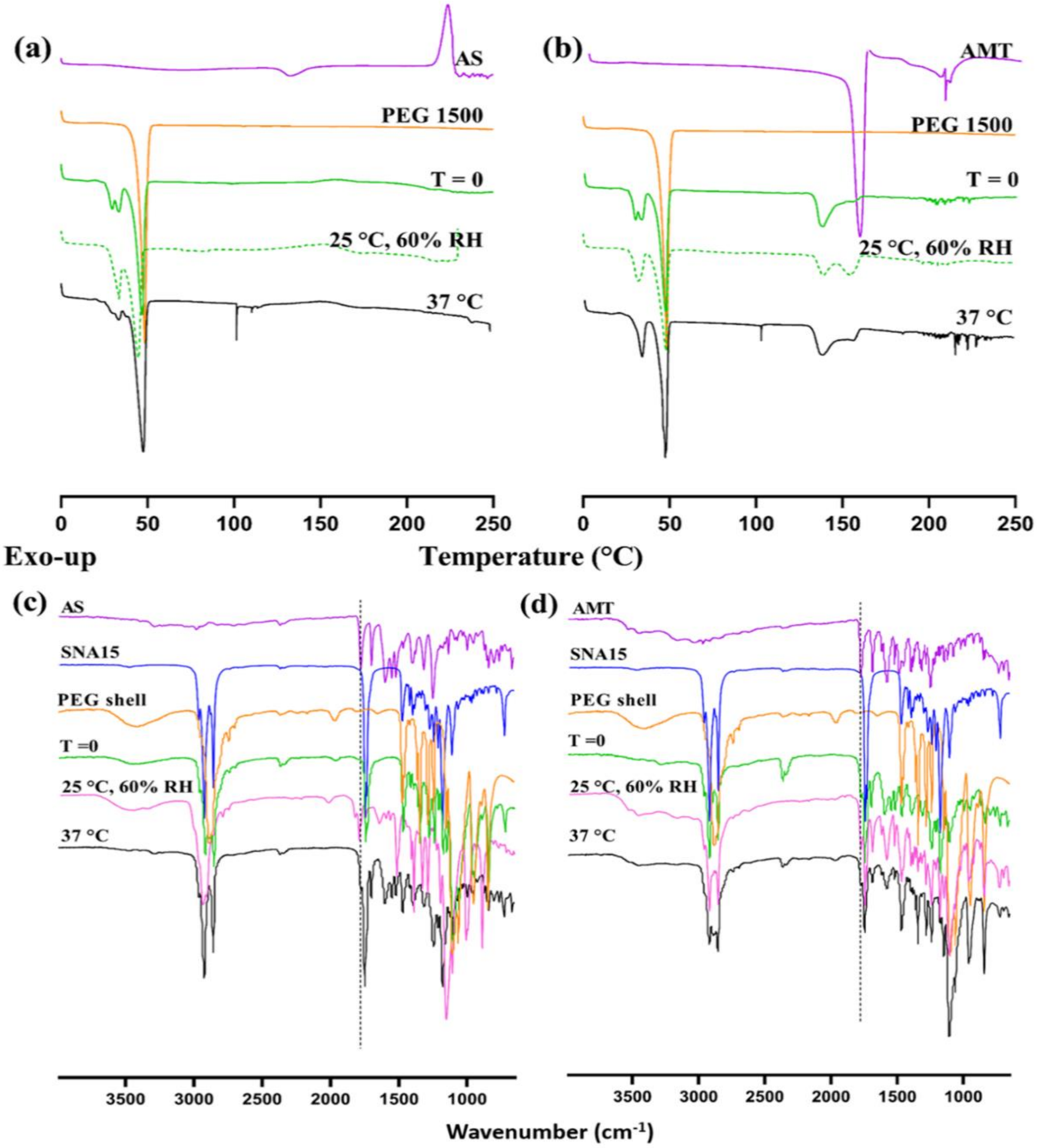

Figure S3. Stability of amoxicillin hollow type suppositories (HTS) and excipients (SNA15 and PEG) at baseline (T=0) and after 3 months, evaluated with DSC (a and b) and FTIR (c and d). Figures (a) and (c) show amoxicillin sodium (AS) HTS and (b) and (d) show amoxicillin trihydrate (AMT) HTS. Suppositories were kept at 25 °C, 60% RH and 37 °C for 3 months. For DSC analysis, samples were heated from 0-250 °C at the rate of 5 °C/min. The vertical dotted line in the FTIR panels (c) and (d) indicate the β-lactam peak at 1772 cm^{-1}.

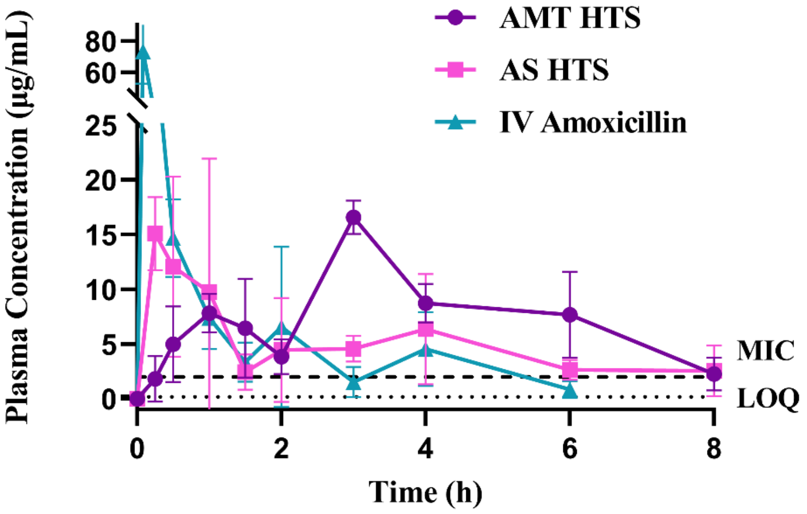

Figure S4. Amoxicillin plasma concentration versus time profile following rectal administration of amoxicillin trihydrate (AMT HTS) and amoxicillin sodium (AS HTS) hollow type suppositories (equivalent to 250 mg amoxicillin) compared with amoxicillin IV injection (at a dose of 100 mg per rabbit). All data have been presented as mean ± SD (n=3 rabbits per treatment). The dashed line shows the minimum inhibitory concentration (MIC) of amoxicillin for *S. pneumoniae* (2 µg/mL), and the dotted line shows the limit of quantification (LoQ) of the HPLC assay.

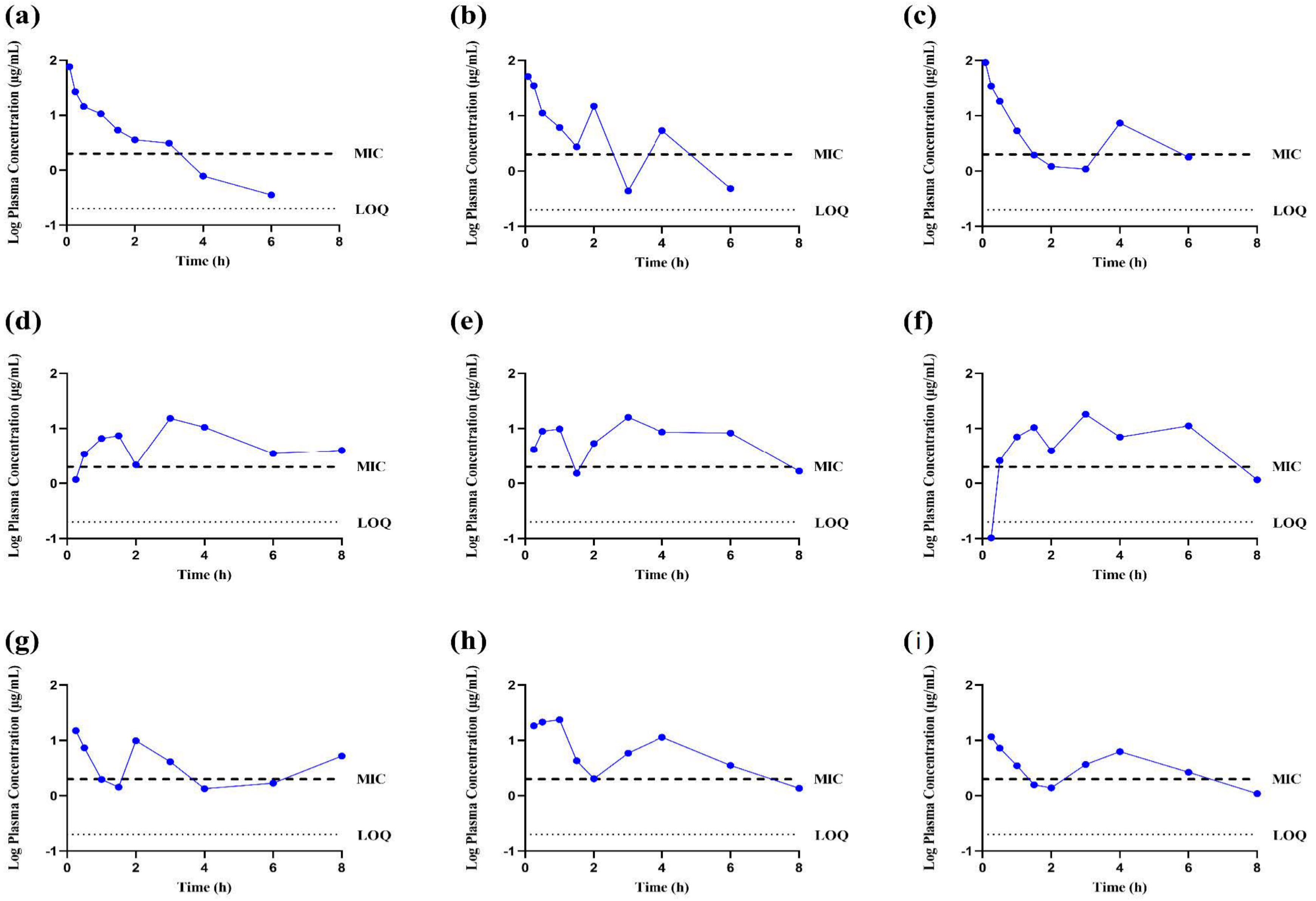

Figure S5. Pharmacokinetic profiles of individual rabbits following the administration of; 100 mg IV solution (a–c), 250 mg amoxicillin trihydrate HTS (d–f) and 250 mg amoxicillin sodium HTS (g–i).
